# Supplementary material for: TSCAN: Pseudo-time reconstruction and evaluation in single-cell RNA-seq analysis
Source: Nucleic Acids Res. 2016 May 13;44(13):e117. doi: 10.1093/nar/gkw430 (PMC4994863; doi:10.1093/nar/gkw430)
Supplement: SUPPLEMENTARY DATA [file supp_44_13_e117__index.html]

TSCAN: Pseudo-time reconstruction and evaluation in single-cell RNA-seq analysis — SUPPLEMENTARY DATA 

# TSCAN: Pseudo-time reconstruction and evaluation in single-cell RNA-seq analysis

## SUPPLEMENTARY DATA

- SUPPLEMENTARY DATA
- SUPPLEMENTARY DATA
- SUPPLEMENTARY DATA
- SUPPLEMENTARY DATA
- SUPPLEMENTARY DATA
