# Supplementary material for: TSCAN: Pseudo-time reconstruction and evaluation in single-cell RNA-seq analysis
Source: Nucleic Acids Res. 2016 May 13;44(13):e117. doi: 10.1093/nar/gkw430 (PMC4994863; doi:10.1093/nar/gkw430)
Supplement: SUPPLEMENTARY DATA [file supp_gkw430_nar-02567-met-g-2015-File005.pdf]

Supplementary Materials

Installation of TSCAN software

TSCAN software package is distributed through GitHub [1] and Bioconductor [2]. The best way to use GSCA is to install and run it on users' own computers. To do so, users have to first download and install R from [3]. Next, users can install the latest TSCAN from GitHub by typing the following commands in R:

```
> if (!require("devtools"))
+     install.packages("devtools")
> devtools::install_github("TSCAN","zji90")
```

Alternatively, one can also install TSCAN from Bioconductor following the instructions in [4]. However, since the Bioconductor only updates its packages twice per year, TSCAN installed from Bioconductor may not be the most up-to-date version.

After installation, one can start the GUI by typing the following commands in R:

```
> library(TSCAN)
> TSCANui()
```

Users are referred to a demonstration video at [5] to learn how to use TSCAN. Users who are familiar with R programming can also run TSCAN using command-line mode. The instructions for using TSCAN as R commands are included in the TSCAN package documentations.

For users who only want to do a one-time analysis, we also created an online web service to help them run TSCAN directly online without the need to install R or TSCAN on their own computers. The link to the web service is provided on the TSCAN homepage at GitHub [1]. The online version, however, can be slow depending on the job load of the web server.

The datasets used in this study can be downloaded from Github [6].

K-means TSCAN

K-means TSCAN is a modified version of TSCAN where mclust is replaced by k-means clustering for clustering cells. All the other procedures in TSCAN

remain the same in k-means TSCAN. In order to determine the cluster number of k-means clustering, we used an approach similar to Figure 1E, with its x-axis changed to the cluster number and its y-axis changed to the proportion of total data variance unexplained by the clusters. More precisely, let $\tilde{\mathbf{E}} = (\tilde{\mathbf{E}}_1, \ldots, \tilde{\mathbf{E}}_N)$ be a matrix containing data for N cells. Each column in the matrix corresponds to a cell. Suppose the N columns are clustered into C clusters. Let $\bar{\mathbf{E}}^{(k)}$ denote the mean of the kth cluster, and let $\bar{\mathbf{E}}$ be the mean of all columns. Let $C(i)$ denote the cluster membership of the ith cell. The total data variance is defined as $SST = \sum_{i=1}^{N} \left\| \tilde{\mathbf{E}}_i - \bar{\mathbf{E}} \right\|^2$ where $\|.\|$ represents l^2 norm. The variance unexplained by the cluster structure is defined as $SSW = \sum_{k=1}^{C} \sum_{i:C(i)=k} \left\| \tilde{\mathbf{E}}_i - \bar{\mathbf{E}}^{(k)} \right\|^2$. The proportion of total data variance unexplained by the cluster structure is SSW/SST. The codes for k-means TSCAN are provided at [7].

Waterfall, SCUBA and Wanderlust for HSMM, LPS and qNSC data

The codes of Waterfall, SCUBA and Wanderlust for analyzing HSMM, LPS and qNSC data are provided at [7].

References

[1] TSCAN home page on Github.
https://github.com/zji90/TSCAN

[2] Bioconductor.
http://www.bioconductor.org/install/

[3] R-project.
http://www.r-project.org/

[4] TSCAN Bioconductor page.
http://www.bioconductor.org/packages/release/bioc/html/TSCAN.html

[5] TSCAN Demo Video.
https://www.youtube.com/watch?v=zdcBAVe1GBE

[6] TSCAN datasets on Github.
https://github.com/zji90/TSCANdata

[7] K-means TSCAN and Waterfall codes for HSMM, LPS and qNSC data.
https://github.com/zji90/TSCANdata/archive/master.zip

List of Supplementary Tables

- Supplementary Table 1 (xlsx format): The correspondence between sample identifiers and data collection time in the HSMM and LPS data. The table has multiple sheets, one for each dataset.
- Supplementary Table 2 (xlsx format): Comparison of functionalities of different single-cell analysis methods.
- Supplementary Table 3 (xlsx format): Cell orderings produced by different methods for each dataset. The table has multiple sheets, one for each dataset and analysis.
- Supplementary Table 4 (xlsx format): Gold standard differential genes for each dataset. The table has multiple sheets, one for each dataset.

List of Supplementary Figures

Supplementary Figures 1-5 are provided below.

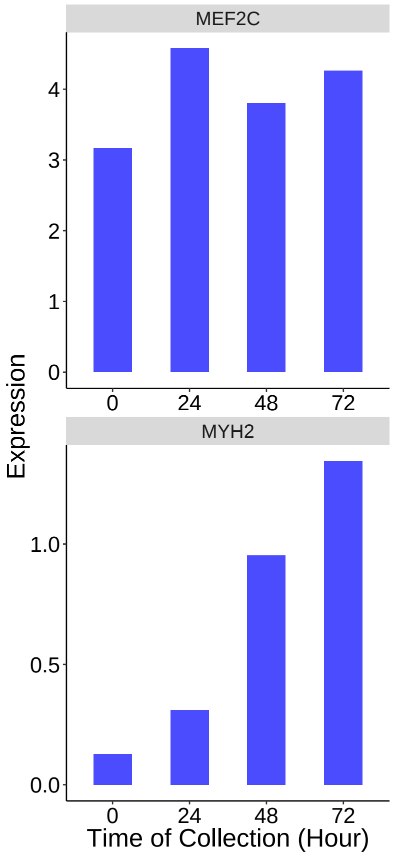

Supplementary Figure 1: Averaged bulk gene expression level for MEF2C and MYH2 in HSMM data.

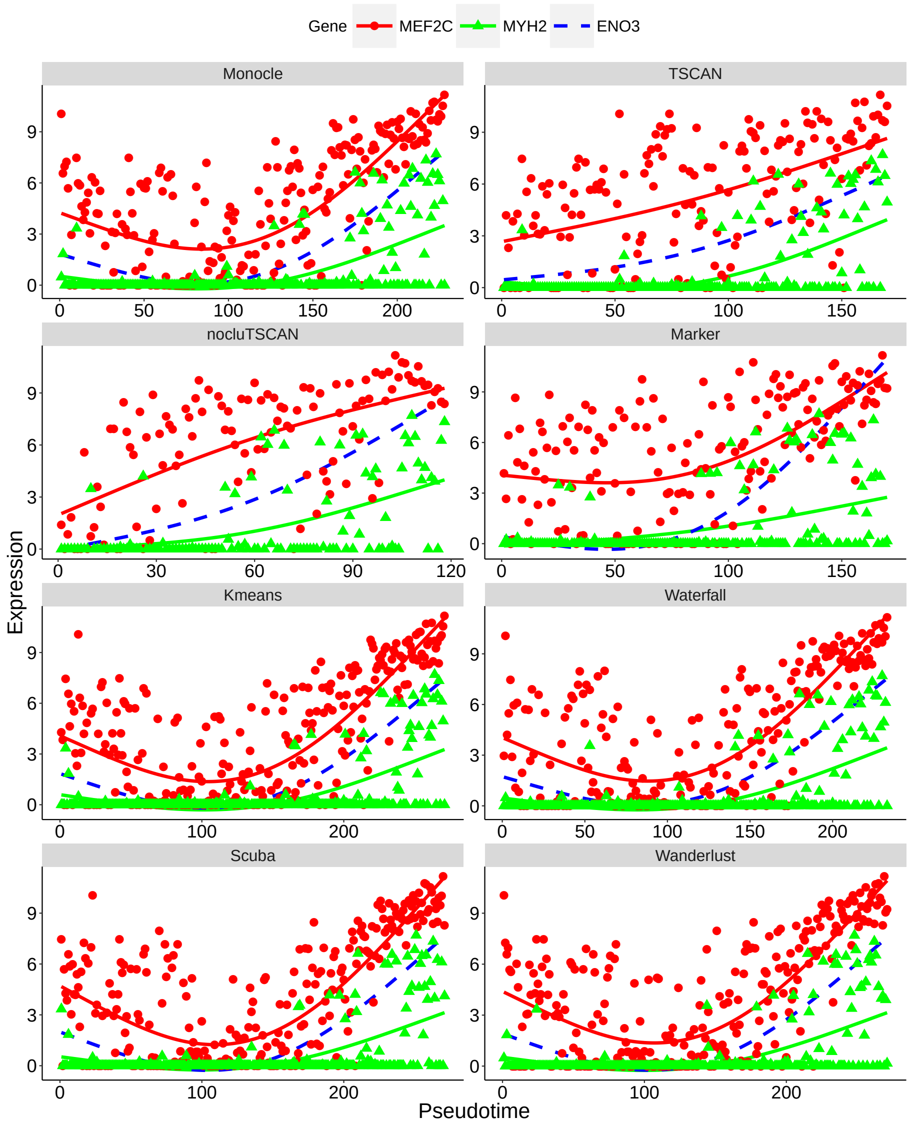

Supplementary Figure 2: MEF2C and MYH2 expression patterns in HSMM dataset where pseudo-time was constructed based on 518 *a priori* chosen genes. MEF2C and MYH2 expression in each cell is plotted as a function of cell order on the analyzed pseudo-time axis. The curves are the fitted GAM function. The dashed curve is the GAM fit for ENO3, the marker used to determine the path direction.

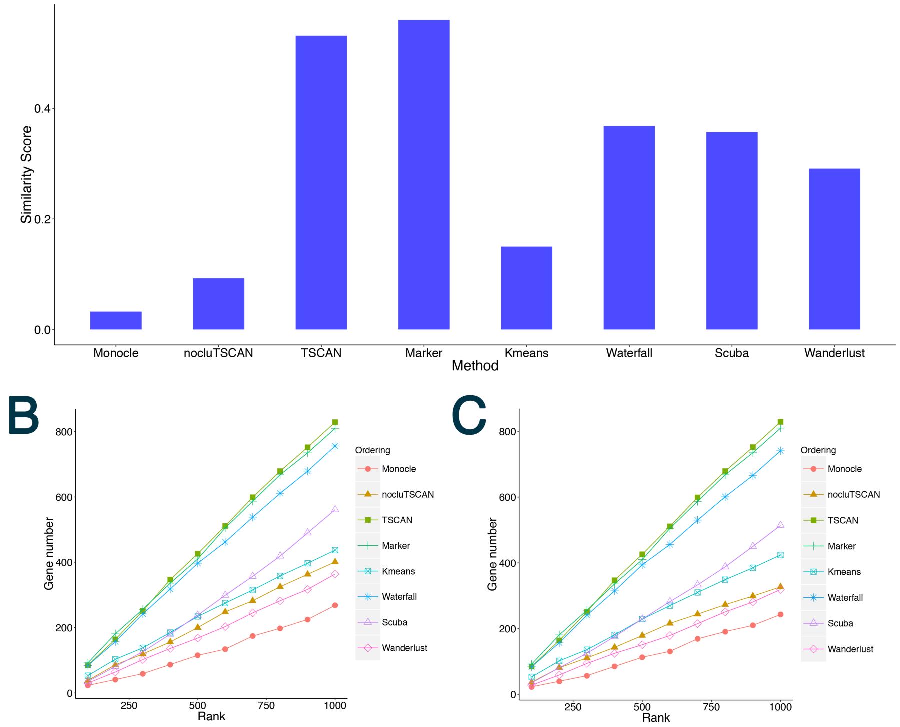

Supplementary Figure 3: Comparing the cell ordering constructed using 518 prior genes and the cell ordering obtained without using these genes in the HSMM dataset. (A) Similarity score between the two orderings for each method. (B) The number of common genes among the top R differentially expressed genes detected by the two cell orderings is plotted as a function of R. (C) The number of common genes with consistent change directions among the top R differentially expressed genes detected by the two cell orderings is plotted as a function of R. In order to determine if a gene has consistent change direction in the two cell orderings, the fitted GAM functions of the gene from the two cell orderings are compared as follows. First, the pseudo-time axes for both cell orderings are linearly scaled to interval [0,1], and the GAM functions are scaled accordingly. Next, values of the GAM functions are extracted at 100 evenly spaced pseudo-time points (i.e., 0.01, 0.02, ..., 1), and then the Pearson's correlation between the two extracted vectors (representing the two GAM functions) is computed. Genes with negative correlation are viewed as inconsistent between the two cell orderings.

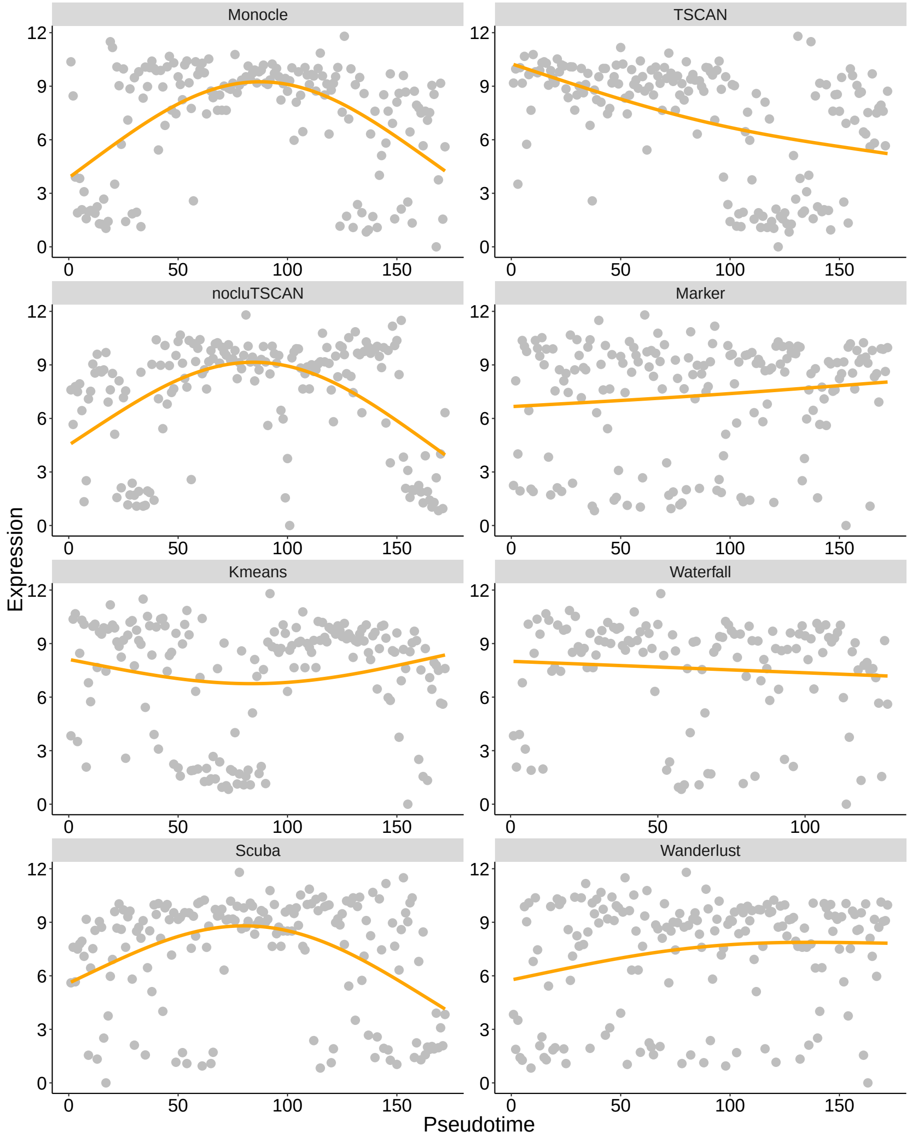

Supplementary Figure 4: SOX9 expression patterns in qNSC dataset. SOX9 expression in each cell is plotted as a function of cell order on the pseudo-time axis. The orange curve is the fitted GAM function.

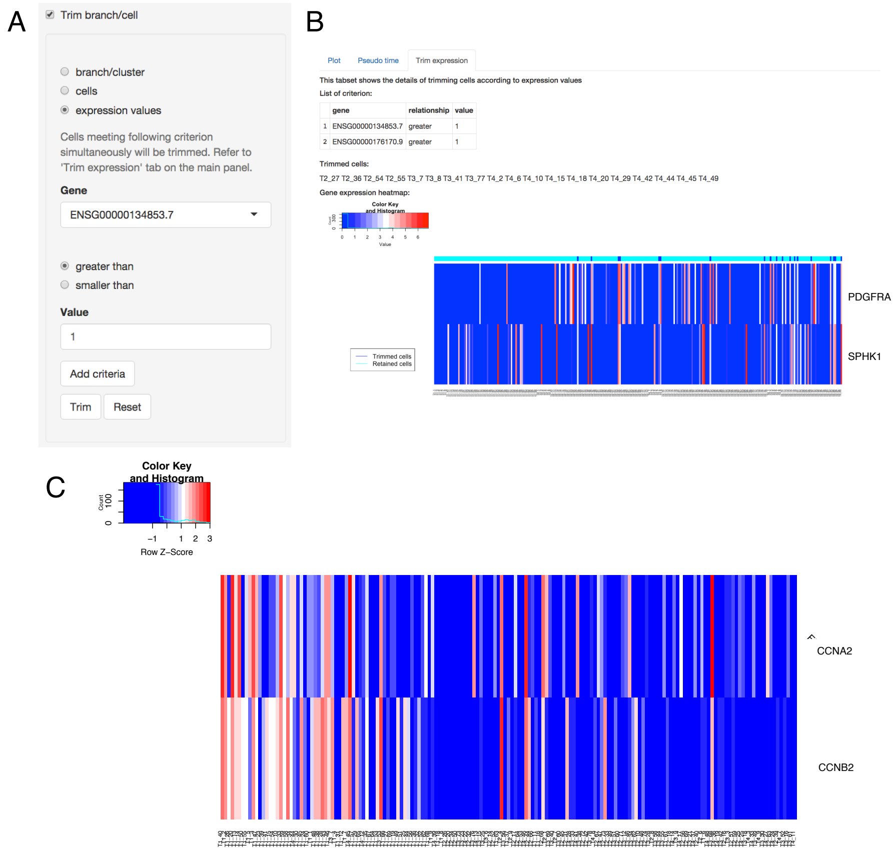

Supplementary Figure 5: Further demonstration of TSCAN GUI. (A) Users can set up trimming criteria by choosing gene names and specifying expression cutoffs. (B) TSCAN excludes cells that meet all trimming criteria. (C) Users can also visualize the expression of specified genes along pseudo-time as heatmaps.
