# Supplementary material for: TSCAN: Pseudo-time reconstruction and evaluation in single-cell RNA-seq analysis
Source: Nucleic Acids Res. 2016 May 13;44(13):e117. doi: 10.1093/nar/gkw430 (PMC4994863; doi:10.1093/nar/gkw430)
Supplement: SUPPLEMENTARY DATA [file supp_gkw430_nar-02567-met-g-2015-File007.xlsx.html]

Oxford Journals | Science & Mathematics | Nucleic Acids Research

We use cookies to enhance your experience on our website. By continuing to use our website, you are agreeing to our use of cookies. You can change your cookie settings at any time. Find out more

3hWaciBYRk30rSOQ7UOpP6viAxsZnEle true

### Looking for your next opportunity?

Looking for jobs...

## Alerting Services

- Email table of contents
- Email Advance Access
- CiteTrack
- XML RSS feed

## Corporate Services

- Advertising sales
- Reprints
- Supplements

Online ISSN 1362-4962 - Print ISSN 0305-1048

Copyright ©  2016 Oxford University Press

**Oxford Journals**   *Oxford University Press*

- Site Map
- Privacy Policy
- Cookie Policy
- Legal Notices
- Frequently Asked Questions

Other Oxford University Press sites:Oxford University PressOxford Journals ChinaOxford Journals JapanAcademic & Professional booksChildren's & Schools BooksDictionaries & ReferenceDictionary of National BiographyDigital ReferenceEnglish Language TeachingHigher Education TextbooksInternational Education UnitLawMedicineMusicOnline Products & PublishingOxford Bibliographies OnlineOxford Dictionaries OnlineOxford English DictionaryOxford Language Dictionaries OnlineOxford Scholarship OnlineReferenceRights and PermissionsResources for Retailers & WholesalersResources for the Healthcare IndustryVery Short IntroductionsWorld's Classics
